# Sharing the Load: Amish Healthcare Financing

**DOI:** 10.3390/healthcare4040092

**Published:** 2016-12-14

**Authors:** Kristyn Rohrer, Lauren Dundes

**Affiliations:** 1 Department of Sociology, Kutztown University of Pennsylvania, Kutztown, PA 19530, USA; krohr501@live.kutztown.edu; 2 Department of Sociology, McDaniel College, Westminster, MD 21157, USA

**Keywords:** Amish, Obamacare, healthcare, insurance, exemptions, Amish Hospital Aid, alms, community

## Abstract

When settling healthcare bills, the Old Order Amish of Lancaster County, Pennsylvania rely on an ethos of mutual aid, independent of the government. Consonant with this philosophy, many Amish do not participate in or receive benefits from Social Security or Medicare. They are also exempted from the Affordable Care Act of 2010. This study expands the limited documentation of Amish Hospital Aid, an Amish health insurance program that covers major medical costs. Interview data from 11 Amish adults in Lancaster County depict how this aid program supplements traditional congregational alms coverage of medical expenses. The interview data delineate the structure of the program, its operation, and how it encourages cost containment and community interdependence. The manner in which the Amish collaborate to pay for medical expenses provides a thought-provoking paradigm for managing health care costs.

## 1. Introduction

### 1.1. Purpose of Study

This study amplifies the limited documentation of Amish Hospital Aid (established in 1969) that helps Amish members pay for health care. Published reports of their unique system can also supplement understanding of the Amish whose evolving code of rules and conduct (the *Ordnung*), varies by subgroup and lacks systematic or explicit written rules. Specific information about the structure of Amish Hospital Aid and its role in how Amish pay for healthcare can inform discussions of how to innovate mainstream US healthcare.

### 1.2. Background

Since 1965, members of religious groups that conscientiously oppose Social Security benefits may apply for an exemption from the self-employment tax (according to the Medicare segment of the Social Security Act). The exemption applies to Old Order Amish, and other religious groups that conscientiously object to insurance, if the sect has been in existence since 31 December 1950 [1]. Amish are part of the “Plain” community, a term that distinguishes Amish and other Anabaptists from outsiders who are called English, fancy people, or white folk. Amish people, whose origins date back to the Anabaptist movement that began in Zurich, Switzerland in 1525, now live in the United States and Ontario, Canada. (The last Amish church in Europe closed in 1937 [2]). Since the Amish first began to immigrate to North America in the 1730s, their population grew to 123,000 by 1991 and to 308,000 in 31 U.S. states [2,3]. Members of exempted religious groups—including the Amish—also must have a reasonable means of caring for their own elderly or dependent members, obviating the need for retirement communities or nursing homes, when each family takes care of its own [4,5].

Amish commonly believe that commercial insurance plans undermine the religious duty of community accountability [2]. Their sense of community is strengthened by the belief that most modern technology brings a worldliness that detracts from their lifestyle. Amish are increasingly likely to use telephones (even cell phones), while televisions, bicycles, gas-powered tractors, and owning vehicles are still forbidden [6].

The 1965 exemptions allow self-employed Amish to avoid paying the combined employee and employer Social Security tax for religious reasons (IRS *Form 4029: Application for Exemption From Social Security and Medicare Taxes and Waiver of Benefits* is for followers of established religious groups that are “conscientiously opposed to accepting benefits of any private or public insurance that makes payments for the cost of medical care; or provides services for medical care”). Amish who work for Amish-owned employers were granted the same exemption in 1988 [7,8]. Amish employed by businesses that are not owned and operated by Amish, however, depend on a different exception—one for individuals who are part of healthcare-sharing ministries [9].

The government describes a health care sharing ministry as “a tax-exempt organization whose members share a common set of ethical or religious beliefs and share medical expenses in accordance with those beliefs”. In addition, the ministry must reject all types of insurance, including Social Security and Medicare, and must have been in existence and sharing medical expenses continuously since 31 December 1999 [10]. These exceptions do not extend to any other taxes, such as real estate taxes, state and federal income taxes, county taxes, and sales tax.

The Affordable Care Act of 2010 (Obamacare) similarly allows exemptions from the requirement to obtain health care insurance for certain religious groups. To compensate for their lack of commercial insurance, the Amish turn to their own community [11,12]. Their way of managing medical costs includes Amish Hospital Aid, an insurance program (that predates Obamacare). This aid program—the focus of this study—is relatively unknown, but received more notice after the passage of the Affordable Care Act in 2010. Access to those who administer and use Amish Hospital Aid was facilitated by a Mennonite member of the Lancaster County community (referred to as “male relative” in this paper).

### 1.3. Amish vs. Mennonite

Mennonites and Amish (as well as Hutterites) share the same roots as participants of the Anabaptist Movement, occurring shortly after the Protestant Reformation. The Anabaptists split into different subgroups before settling in North America, though many of them settled within close proximity to one another. In Lancaster County, there are few Amish subgroups. It is almost exclusively Old Order Amish who have remained together under the same doctrine of faith. This contrasts with Mennonite groups and Amish communities in other areas, which have experienced multiple religious splits [8].

There are two major groups of Mennonites: Plain Mennonites and assimilated Mennonites. Plain Mennonites or Old Order Mennonites are often confused with the Amish, as they share many of the same religious beliefs and cultural ideologies. There are numerous different divisions of Plain Mennonites in Lancaster County. Some Plain Mennonites use horse and buggy for transportation (Team Mennonites), while others such as the Black-Bumper Mennonites drive all-black cars (i.e., the chrome, bumpers, etc. are painted black). There are general differences in the guidelines and style of clothing, head coverings, and buggies within the Plain community, distinguishing the Amish from the Plain Mennonites. Mennonites are also permitted to ride bicycles, while the Amish are not. On the other hand, unlike the Amish, Plain Mennonites believe other religious groups (including the Amish and assimilated Mennonites) are not “saved”. Assimilated Mennonites are essentially indistinguishable within mainstream society. They are allowed to wear contemporary clothing, use electricity and new technology, attain higher education, and do not live in community settings (as do the Amish and Plain Mennonites).

## 2. Methods

### 2.1. Access to the Amish

The ability to interview members of the Lancaster County Amish community required special arrangements and stipulations given their reluctance to associate with and trust outsiders, known as “English”. This term refers to the language of outsiders that contrasts with the variant of German (called Pennsylvania Dutch) which Amish speak among themselves (“Dutch” is likely derived from the word *Deutsch* meaning “German”, not Dutch). Since Amish learn English in school, the interviews could be conducted in English.

The first author received assistance from an assimilated Mennonite male relative who has a 16-year career in business and is well known in the Amish community for his integrity. His heritage also provided valuable insight into the Amish culture (e.g., his Mennonite grandfather in retirement refused to accept Social Security income beyond what he had paid into the system) The relative granted permission to include all of the information identifying him in this paper.

The relative offered to approach and help interview contacts in the Old Order Amish community of Lancaster County, PA, USA (the largest Amish community comprising about 35,000 persons) [3]. He was able to advise the authors on factors critical to developing a rapport with Amish interviewees, including norms for appropriate dress (ankle-length skirts for women), the prohibition on taking pictures of the Amish (although photos of their homes are not forbidden), and scheduling norms (e.g., working around the wedding season in mid-October through mid-December, and the inability to schedule interviews more than a few days or hours ahead of time) [13].

### 2.2. Obtaining Interviews

After receiving IRB approval from McDaniel College on 27 October 2015, and in accordance with the rules of the Declaration of Helsinki of 1975, the first author (accompanied by her male relative) interviewed 11 individuals after they signed an informed consent form. The sample consisted of seven men and four women from nine separate households who had experienced major medical expenses or were heavily involved in the Amish Hospital Aid program, including those in administrative positions. The relative specifically recruited interviewees to provide a variety of perspectives, both in administering and receiving Amish Hospital Aid. A majority of the interviewees (8/11) had previously established a relationship with the male relative that facilitated interviewee recruitment, while the remaining respondents were referred to the male relative by initial respondents because of their knowledge of Amish Hospital Aid (either due to having had major medical expenses or administrative involvement in the program). One respondent not previously acquainted with the male relative—an Amish Hospital Aid administrator—sought additional clarification about the purpose of the study before he consented to the interview. The response rate among those approached was 100%.

The individuals were told that the purpose of the study was to collect and publish data documenting how the Amish manage health care needs within their own system (given their exemption to coverage mandated by Obamacare). Ten of the 11 interviewees were members of the Amish Hospital Aid plan at the time of the interviews, although one had only joined Amish Hospital Aid subsequent to a major medical expense. As is normal among the Amish, all of the respondents received a formal education only through 7th or 8th grade.

The first author and her male relative conducted the interviews in the kitchen of interviewees’ homes or in their office/workspaces between 25–27 November 2015. Interview questions concerned their involvement in the program, participation in conventional medical care, and the operation of the Amish Hospital Aid program. Interviews lasted between 30–75 min and were documented by the first author with handwritten notes.

## 3. Results

### 3.1. Traditional Ways of Paying for Health Care

Although much of the information shared during interviews has not been previously documented, existing literature (when available) corroborated the findings. While the focus in the interviews was Amish Hospital Aid (a type of Amish insurance), respondents also discussed traditional ways their community helps with unmanageable healthcare costs that at times operate in tandem with Amish Hospital Aid. One source of handling medical costs that exceed an individual’s ability to pay out of pocket is voluntary donations to congregations, called alms, which are in line with a tradition of sharing burdens in the Amish community [9]. Alms are tithes or offerings donated to the congregation by its members. As with tithes in other Christian churches, members are encouraged to give 10% of their annual income to the Amish church district. Church deacons, who are in charge of both disciplinary and financial matters in the congregation, visit members in need of medical assistance to see how they are faring and then distribute the alms as they see fit.

In the case of more serious injuries, when an individual’s congregation cannot afford to pay the medical bills solely with their own alms funds, Amish congregations may use community collections. Community collections are a form of alms that are gathered from the alms of other Amish congregations in the area. These funds can be requested at the discretion of a deacon. Community collections were used when a man was paralyzed from the waist down after a diving accident. Occasional auctions of donated food, furniture, quilts, and livestock can raise as much as three hundred thousand dollars in one evening to help supplement alms and/or community collection coverage of healthcare bills [8,9]. In extremely rare cases, the government has covered medical costs (e.g., when an uninsured driver hit a buggy).

### 3.2. Implementation of Amish Hospital Aid

Amish Hospital Aid covers only major medical needs. In serious cases (normally when hospitalization is necessary), those who participate in the program contact the treasurer in charge of their district once they know the costs incurred or to be incurred. Those requiring care typically pay the health care provider used, and Amish Hospital Aid then reimburses them. Members who are unable to pay upfront allow the board to make arrangements with the hospital or care facility, in order for the board to pay the provider directly. The first 20% of the bill is expected to be paid by the individual, while the other 80% is covered by the Amish Hospital Aid plan. Those who are unable to pay the first 20% often rely on alms money from their congregation.

### 3.3. How Amish Hospital Aid Manages Medical Costs

When ill, Amish seek treatment at their local hospital and are billed the same as non-Amish. The individual then requests help from the congregation and/or Amish Hospital Aid for any unaffordable medical expenses (e.g., maternity complications, surgeries, head injuries, physical therapy, and geriatric care). The Amish Hospital Aid board also works closely with bill negotiators at different hospitals and facilities, just like commercial or governmental insurance companies, to negotiate discounts for individuals with specific needs [9]. Incentives to provide discounts include the promptness with which bills are normally paid (within 30 days), less paperwork, as well as assurance that the facility will not be sued (since doctors are seen as fallible but autonomous individuals doing their best) [14]. Typically, participants of Amish Hospital Aid receive a discount slightly above Medicare rates, although each medical provider has its own particular discount. Not all care facilities offer a discount for members of the Amish community, however. Hospitals sometimes refuse to consider lower rates beyond existing negotiated rates with government or commercial insurance companies [2]. On the other hand, health care facilities like the Clinic for Special Children provide pediatric care, especially for genetic disorders and syndromes (in Strasburg, Pennsylvania) for Amish and Old Order Mennonites, who may travel a great distance to reach the facility in order to receive state-of-the-art care and save money on treatment. The Clinic offers substantial reductions in health care costs by such means as lowering the price of testing, gauging when expensive treatment is warranted, and sometimes by devising treatments that prevent costly disability [15,16]. (A short video (3:25) about the clinic is available in a link in a *Wall Street Journal* article [17].)

### 3.4. Restrictions in Amish Hospital Aid Plan Coverage

The Amish Hospital Aid Plan includes limitations in its coverage, namely because it covers only major medical costs (hence the word “hospital” in its name). This discourages routine and preventive medical services—particularly by family doctors—and is a source of discontent for some interviewees. In addition, Amish Hospital Aid does not cover physical disability costs, such as those for Cerebral Palsy. Another Amish-run organization, *Disability Relief Aid*, covers costs for necessary items such as wheelchairs, ramp installations, and special bathroom installations, in addition to supplying an annual check to help with personal care costs. As with alms, *Disability Relief Aid* is funded by community donations.

Neither Amish Hospital Aid nor congregational alms funding cover health care needs that result from prohibited activities within the Amish community. One interviewee mentioned an incident that occurred with a teenage boy in her congregation who was injured in a snowmobiling accident. The use of motor vehicles (e.g., cars, tractors, snow mobiles) is strictly forbidden in Old Order Amish culture. Even though the boy was not yet baptized into the Amish congregation (and therefore still under the aegis of his parents), the deacon would not provide alms money to help pay for his hospital care. The boy’s parents also participated in the Amish Hospital Aid plan, which likewise did not provide financial support, even though since he was under 18, he was covered under his parents’ plan.

### 3.5. Extent of Participation in Amish Hospital Aid

While members of the Amish community are not required to participate in Amish Hospital Aid and may just rely on alms, a growing number of Amish want the extra security provided by Amish Hospital Aid. Interviewees cited an estimated 7000–8000 participants in Lancaster County or a participation rate of approximately 30%.

The Amish National Steering Committee (the Plain Community’s liaison to the national government) had new work as a result of Obamacare. In 2014, the Committee instructed each head of household in the Amish Community to sign forms from Health and Human Services (HHS) to extend their exemption to Obamacare. HHS provided each deacon with the forms to be distributed to members of their church congregations. Members gathered at the usual Sunday service, with men sitting in groups of ten at round tables. One man explained the meaning of the forms to the others before they all signed the forms. In response to Obamacare, participation in Amish Hospital Aid increased by 10% from 2014 to 2015.

### 3.6. Hierarchy of Amish Hospital Aid Administration

The Amish Hospital Aid plan is run by an all-male board (consisting of a chairman, vice chairman, and four treasurers). Each treasurer is in charge of the funds for approximately 50 congregations. The leaders appoint a Committee Man for each congregation to act as a liaison between the members and the administration. The Hospital Aid committee (including an estimated 200 Committee Men and the 6 board members) meets annually to discuss the program. The entire committee participates in voting, with board members holding six-year terms with no limit on reelection. Current Committee Men are candidates for members of the board, by recommendation. Those with Amish Hospital Aid typically contact their treasurer once they receive their medical bill. In some cases, the treasurer actually contacts whoever receives the bill. Since news travels fast within the Amish community, if someone has been injured or hospitalized, members of the congregation will know, including the individual’s treasurer.

All members of the Committee are men, since there are no women in administrative positions in the Amish Hospital Aid program, as is consistent with organizations in the Amish community. However, women are allowed and often encouraged to have their own personal businesses (selling quilts, fabrics, baked goods, etc.), and are sometimes in charge of the family finances instead of their husbands.

### 3.7. Role of Unpaid Administrators

The Amish system of paying health care bills has existed very informally, driven partly by the notable fact that administrators are not paid for their time. “Remarkably, common to all these aid programs is their ability to address major needs without bureaucratic red tape, paid employees, underwriters, offices, computers, threat of lawsuits, or profits” [2] (p. 188). This system contrasts with administrative expenses among US private insurers who spend 12% of their budget on costs like claims processing, marketing, and general overhead [18]. Administrative costs at US hospitals are even higher, at 25% of total hospital expenditures [19]. All respondents in this study were aware of and supported a lack of paid administrators. They offered comments such as, “If it ain’t broke, don’t fix it”.

### 3.8. Cost of Amish Hospital Aid

Members pay a flat rate per person on a monthly basis. The monthly cost of $125 per individual is exactly the same as what was reported in a *Wall Street Journal* article seven years prior to data collection for this study [17]. A married couple pays $250 per month; however, all of their children until the age of 18 would also be covered by the $250 payment. One interviewee who complained about the high monthly cost (mentioned above) speculated that the “English” (i.e., non-Amish people) “probably don’t pay nearly that much”. This interviewee was very surprised to hear that the costs for non-Amish who use conventional insurance are in fact significantly higher than what he was paying for Amish Hospital Aid (although the type of coverage provided by conventional insurance varies).

The funds are payable to the Committee Men on the first day of every month. Members tend to be very punctual with their payments and are contacted (sometimes by phone) if their check does not arrive by the due date. The Committee Man then transfers the funds collected to the district’s treasurer who then transfers them to the bank for later dispersal, as needed.

### 3.9. Complementary Care Used to Minimize Conventional Care

Respondents were asked about ways they stayed healthy and minimized the need for health care. They mentioned attention to good nutrition and exercise, including their vigorous farm work. In addition, unmarried Amish youth often participate in sports such as softball and volleyball. They frequently take natural vitamins and probiotics in order to minimize health problems, such as Vitamin C for a bad cough. Home remedies are typically used for less serious ailments (such as colds, minor burns, and infections, etc.), and replace visits to a family doctor. Remedies mentioned included a combination of herbs and whiskey (known as tincture) for a sore throat, burdock for burns, and charcoal for infections, many of which can be found in an often-mentioned book, *Be Your Own Doctor* [20] (a local health food store *Miller’s Natural Foods* located in Bird-in-Hand, PA supplies many Amish families in the Lancaster County area with natural vitamins and supplies for home remedies).

It is common for individuals to follow their family’s conventions in their inclinations to go to a medical doctor. Nonetheless, there is a lot of variability in opinion: one respondent stated that he would go to the doctor if he had a cold he could not shake, while his wife said that for similar problems, she prefers home remedies. The continued used of complementary medicine in the Amish community, however, does not preclude mainstream medical treatment (perhaps in part an acknowledgement of the limits of complementary care which is not always beneficial).

### 3.10. Willingness to Seek Conventional Medical Care

Amish should not be confused with other groups loathe to seek modern health care for themselves or their children (e.g., [21]). Chiropractic care (including for infants) and phenology are especially popular [20]. Despite stereotypes that the Amish are Luddites—perhaps assumed because of their use of the horse and buggy—Amish are generally very willing to take advantage of the most cutting-edge technology to help remedy their children’s maladies, no matter the cost to them personally [17]. They avoid only technology that they believe detracts from their relationship with God, or family and community life.

Many interviewees expressed a willingness to benefit from modern health care, without moral (if not financial) reservations. Many indicated that they would seek conventional care: “if something happens” or “whenever we need it”. Additional related comments included: “If it’s a doctor thing, it’s a doctor thing” and “If something looks fishy, check it out”. Some Amish only visit their family doctor for an annual check-up, while others wait until an injury occurs. Most Amish praised the health care they had received, especially doctors who were good listeners and willing to “cater to the Plain community” by not necessarily thinking like a medical doctor (e.g., allowing Amish families to practice home remedies). One woman whose young daughter had a genetic disorder that required her to be in and out of the hospital for the first few months of her life spoke highly of the extra care that her doctors from the above-referenced Clinic for Special Children provided to the family, including monthly visits directly to their family farm to check on her daughter.

The Amish approach to seeking conventional medical care also takes religion into account:

“Amish people are more willing to stop interventions earlier and resist invasive therapies than the general population because, while they long for healing, they also have a profound respect for God’s will. This means taking modest steps toward healing sick bodies, giving preference to natural remedies, setting common-sense limits, and believing that in the end their bodies are in God’s hands”.

[2] (p. 336)

### 3.11. Why Respondents Support Amish Hospital Aid

Interviewees referred to Amish independence in explaining the need for having their own system for health care payments. Comments included: “The Amish community is not in favor of government hand-outs”, “Amish prefer to take care of their own”, “Obamacare requires too much government involvement”, “The less government the better”. One respondent opined that “Obamacare commandeers freedom of choice” and contrasts with the ability of members of the Amish Hospital Aid plan to choose whichever medical facility best suits their needs.

Respondents also mentioned the benefits of assisting others and helping others while helping themselves: “We appreciate the privilege to take care of our own people”. Their belief that a communal approach to covering health costs is morally right is consistent with their strong commitment to community members looking after one another.

### 3.12. Divisions in the Amish Community

The Amish Hospital Aid program is not without challenges, however, as more conservative Amish (who tend to reside in the southern areas of Lancaster County) are less likely to participate in the program, reflecting a well-known north–south divide in Lancaster County (roughly represented by Route 30). One southern respondent who objected to Amish Hospital Aid explained that “Plainer Amish don’t use it” and perceived it as, “the rich helping the rich”. Northern Lancaster County Amish (e.g., those living in and around Intercourse) tend to be more affluent than their more conservative southern neighbors living in towns like Quarryville. Relative to much more conservative Amish in other parts of Pennsylvania, however, the difference between northern and southern Lancaster County Amish is much less prominent.

Some of the more conservative Amish see the Amish Hospital Aid plan as inappropriately progressive and institutionalized. Many believe it detracts from neighbors helping each other completely voluntarily (with its set monthly fees, etc.) and view it as taking away from donations to the alms fund, a belief that remains to be substantiated. These individuals are more apt to approve only of the traditional Amish alms support (akin to church tithing) for members’ medical needs [2]. Part of the appeal of alms is the reliance on voluntary donations, which bears no resemblance to standard health insurance. Nonetheless, the extent of participation in Amish Hospital Aid is growing in southern settlements of Lancaster County.

Some northern Amish prefer the Amish Hospital Aid plan because alms are sometimes considered “poor money”, making wealthier Amish feel guilty if they receive financial support from the congregation that is traditionally meant for those who are less financially stable. The question of wealth is complicated for Amish who have assets in land but few liquid assets, such as cash or things they could sell quickly to pay a medical bill.

One respondent frowned on wealthier Amish whom he believed would be exploiting alms if they have significant funds tied up in land. He gave a hypothetical example of someone who owned a couple of farms who had a bad accident. He claimed that the congregation would not expect an individual to sell one of their farms to make the payments, so the deacon would provide alms money. He added, “I’d be a hypocrite for having two farms and taking money from the church”.

This hypothetical example has similarities with the publicized case of Jesse Martin, an Old Order Mennonite farmer from Denver, Pennsylvania who received national news coverage in 2008 because of his struggles to pay for healthcare. Because Martin did not sell any part of his two valuable farms, he was unable to pay bills totaling hundreds of thousands of dollars for nine of his 11 children who had serious genetic disorders including Hirschsprung disease and maple syrup urine disease [17]. In cases where there are many high medical bills, even a combination of personal resources, alms, and Amish Hospital Aid can be insufficient [17]. Nevertheless, these cases are the exception. According to a health survey of Lancaster County Old Order Amish (response rate: 49%), their system is adequate: only 16% of Amish surveyed had delayed care in the past 12 months (most commonly because of the expense), and only 13% (vs. 18% of county residents) rated their health as fair or poor [22].

### 3.13. Limitations

This research relies on a small but select sample of individuals in a limited geographical area who were able and willing to discuss the administration of Amish Hospital Aid and its advantages and shortfalls. Although the sample was biased based on the male relative’s business connections, individuals were selected for their knowledge and insight on the topic, as well as a commitment to providing new in-depth and candid information about the Amish. It is important, however, to acknowledge that the small sample size limits the generalizability of these findings, despite the thoughtfulness of respondents’ answers. Data from a random sample would likely have revealed different reactions to Amish Hospital Aid, although a random sample would have yielded a significantly lower response rate from those outside of the social network of the community shared by Amish and Mennonites. In addition, given that the data provided were confidential, but not anonymous, some interviewees may have limited what information they disclosed during the interview. Nevertheless, because the main goal of the study was to understand the structure of Amish Hospital Aid, interviewees may have felt more comfortable than if the research were designed to assess the program’s popularity or effectiveness.

## 4. Discussion

This paper describes a unique community-based approach to healthcare financing. The Amish have achieved and sustained a large measure of self-sufficiency in their own system for managing costs that reflects the spirit of mutualism. The Amish approach provides a stark contrast to the current mainstream healthcare environment, where there is significant federal government control over healthcare decision-making and a pressing need to curtail spending.

The Amish also provide a small-scale example of healthcare rationing by implementing a program that covers only major medical needs. By limiting coverage, they have devised a manageable system bolstered by a strong sense of personal and group responsibility. Because members of Amish Hospital Aid can, if necessary, seek help to pay their 20% share of a bill through alms, they must be cognizant of their standing in the community, since coverage of an individual’s share is subject to review. This facet of the system reinforces Amish inter-connectedness, as they face healthcare challenges collectively, mindful that judicious use of health care resources—including preventative measures—benefit the community.

In the Amish system, individuals are more likely to understand that it is impossible to cover all medical desiderata. In contrast, in the mainstream healthcare system, there is no way for patients to see that spending money on one individual perforce reduces the amount that can be spent on others. Amish insight into this equation helps to provide an incentive on an individual level to limit care or costs that are otherwise very abstract. Collaborative efforts by the Amish to manage healthcare costs could inspire new ways of thinking about containing costs while building community.

## 5. Conclusions

The Amish norm of reciprocal assistance without government interference is the basis for a system of paying for healthcare that builds on existing resources while limiting coverage. This article elucidates the unique way that a sample of Old Order Amish of Lancaster County manage healthcare financing by providing never-before reported information about the organization and administration of Amish Hospital Aid. This information was obtained through the assistance of a trusted Mennonite community liaison, imperative to elicit the level of candor needed to learn the kinds of details contained in the interview data presented in this article. Although the sample size is small, interviewees include persons very familiar with the inner workings of Amish Hospital Aid. Documenting the unorthodox manner in which the Amish community collaborates in managing healthcare costs could inform innovative alternatives to mainstream healthcare financing.
